# Influenza vaccination in patients with end-stage renal disease: systematic review and assessment of quality of evidence related to vaccine efficacy, effectiveness, and safety

**DOI:** 10.1186/s12916-014-0244-9

**Published:** 2014-12-19

**Authors:** Cornelius Remschmidt, Ole Wichmann, Thomas Harder

**Affiliations:** Robert Koch Institute, Immunization Unit, Seestrasse 10, 13353 Berlin, Germany

**Keywords:** Dialysis, Effectiveness, End-stage renal disease, GRADE, Influenza, Systematic review, Vaccine

## Abstract

**Background:**

Vaccination against influenza is recommended in patients with end-stage renal disease (ESRD). However, so far, no systematic review has summarized the available evidence on the effectiveness and safety of influenza vaccination in this patient group.

**Methods:**

We conducted a systematic review and meta-analysis and assessed the quality of evidence using the GRADE methodology. We searched MEDLINE, EMBASE, Cochrane Library databases, ClinicalTrials.gov, and reference lists for studies on efficacy, effectiveness, and/or safety of seasonal influenza vaccination in patients with ESRD receiving dialysis. All reported clinical outcomes were considered, including all-cause mortality, cardiac death, infectious death, all-cause hospitalization, hospitalization due to influenza or pneumonia, hospitalization due to bacteremia, viremia, or septicemia, hospitalization due to respiratory infection, ICU admission, and influenza-like illness.

**Results:**

Five observational studies and no randomized-controlled trial were identified. In four studies, risk of bias was high regarding all reported outcomes. Strong residual confounding was likely to be present in one study reporting on three outcomes, as indicated by significant protective effects of vaccination outside influenza seasons. Therefore, the statistically significant protective effects on all-cause mortality (vaccine effectiveness (VE), 32%; 95% CI, 24–39%), cardiac death (VE, 16%; 95% CI, 1–29%), hospitalization due to influenza or pneumonia (VE, 14%; 95% CI, 7–20%), ICU admission (VE, 81%; 95% CI, 63–86%), and influenza-like illness (VE, 12%; 95% CI, 10–14%) have to be taken with caution. According to GRADE, the quality of the body of evidence was considered very low for all outcomes. No study reported on laboratory-confirmed influenza virus infections or on safety endpoints.

**Conclusions:**

Evidence on the protective effects of influenza vaccination in patients with ESRD is limited and of very low quality. Since VE estimates in the available literature are prone to unmeasured confounding, studies using randomization or quasi-experimental designs are needed to determine the extent by which vaccination prevents influenza and related clinical outcomes in this at-risk population. However, given the high rates of health-endangering events in these patients, even a low VE can be considered as sufficient to recommend annual influenza vaccination.

**Electronic supplementary material:**

The online version of this article (doi:10.1186/s12916-014-0244-9) contains supplementary material, which is available to authorized users.

## Background

Worldwide, about 1.9 million patients undergo renal replacement therapy (hemodialysis, peritoneal dialysis, kidney transplantation) due to end-stage renal disease (ESRD) [1]. Although rates of incident ESRD have decreased in some countries, the burden of ESRD is increasing globally [2], mainly driven by an increase in the prevalence of major risk factors such as diabetes and hypertension [3]. Due to multifactorial causes, patients with ESRD have an impaired innate and adaptive immune system, including defects in complement activation and B- and T-cell function [4-6]. This functional abnormality contributes to higher incidences and severe courses of infectious diseases [7,8]. For example, pulmonary infection-related mortality is up to 10-fold higher in ESRD patients compared with the general population [9]. To reduce influenza disease burden among these patients, the World Health Organization and many national immunization technical advisory groups recommend annual vaccination against seasonal influenza [10-13].

Although studies suggest that immunogenicity might be reduced among ESRD patients [14], antibody levels regarded as protective have been found in 53 to 90% of dialysis patients [15,16]. However, it is unclear to which degree these antibody levels finally translate into the prevention of clinical outcomes. A recent methodological study indicated that no systematic review has been published thus far on the efficacy, effectiveness, and safety of seasonal influenza vaccination in patients with ESRD receiving dialysis [17]. We therefore performed a respective systematic review and meta-analysis and used the methodology suggested by the Grading of Recommendations Assessment, Development and Evaluation (GRADE) working group to rate the quality of the body of evidence for each outcome.

## Methods

### Search strategy and selection criteria

This systematic review was performed according to the Preferred Reporting Items for Systematic Reviews and Meta-analysis (PRISMA) statement [18]. The literature search was performed irrespective of study design (experimental and observational) and publication language. The electronic databases searched were MEDLINE, EMBASE, Cochrane Central Register of Controlled Trials, Cochrane Database of Systematic Reviews, and Database of Abstracts of Reviews of Effects (date of last search: 07 May 2014). The complete search strategy is shown in Additional file 1. In addition, we searched for unpublished or ongoing studies in ClinicalTrials.gov. Electronic searches were complemented by manually searching all reference lists of identified studies and reviews for additional studies.

Studies had to meet the following *a priori* defined inclusion criteria: i) original report on efficacy, effectiveness, and/or safety of vaccines against seasonal influenza in patients with ESRD receiving either hemodialysis or peritoneal dialysis, and ii) control participants had to be either unvaccinated or must have received placebo. We excluded studies in which participants in the intervention arm had received more than one influenza dose in a given season.

### Data extraction and risk of bias assessment

Two reviewers (CR and TH) independently screened titles and abstracts to identify potentially eligible studies which were then reviewed as full text. Disagreements were resolved by discussions until consensus was achieved. From eligible studies, two independent investigators (CR and TH) extracted study characteristics and assessed risk of bias, using standardized forms. Disagreements between extractors were resolved by discussion. From each study, the following information was extracted: study design, country, study period, data source(s), population size, inclusion and exclusion criteria for participants, age at vaccination, sex, mean duration on dialysis, ethnicity, duration of follow-up, reported comorbidities, source of information on vaccination, vaccine used, circulating influenza strains, match/mismatch between vaccine and circulating strain, relative risk (RR), odds ratio (OR) or hazard ratio (HR) for defined outcomes, risk difference (RD), confounder-adjusted estimates, confounders considered, and control period (off-season) estimates. We used the tool developed by the Critical Appraisal Skills Programme [19] to assess risk of bias in the included studies. According to the suggestions by the Cochrane Collaboration [20], we made this assessment separately for each outcome and expressed the result as a considered judgment, using the categories “high risk of bias”, “low risk of bias”, and “unclear risk of bias”.

### Assessment of the quality of a body of evidence

For each outcome, the quality of the respective body of evidence (i.e., across all included studies) was assessed using the GRADE methodology [21,22]. According to GRADE, evidence on the effects of an intervention is categorized into four levels of quality: very low, low, moderate, and high. Bodies of evidence from randomized controlled trials (RCTs) start as high quality evidence, whereas those from studies with other designs (observational studies) start as low quality evidence. According to a set of predefined criteria, evidence quality can be increased or decreased. Further details on GRADE can be found elsewhere [21,22]. In order to assess the best available evidence, we used the results of the confounder-adjusted analyses to determine GRADE evidence quality.

### Data synthesis and statistical analysis

RRs, ORs, HRs, and RDs and corresponding 95% confidence intervals (95% CIs) were either calculated or extracted directly from the publications. Vaccine effectiveness (VE) was calculated as 1 – RR × 100. To express the number of individuals needed to be vaccinated to prevent one case of a particular outcome, we calculated the number needed to vaccinate (NNV) as 1/RD.

Where data from more than one study for a given outcome were available, we performed a meta-analysis, using a random-effects model to account for heterogeneity. If a single study reported data from more than one season, these point estimates were pooled prior to meta-analysis. I^2^ was used to quantify the extent of heterogeneity. Confounder-adjusted in-season estimates were pooled if they were adjusted at least for age, sex, and comorbidities. Since two studies covered patients that might be different compared to the patients on continuous hemodialysis included in the remaining studies, namely patients with newly diagnosed ESRD [23] or patients on peritoneal dialysis [24], we conducted a sensitivity analysis by stepwise excluding data from i) patients on peritoneal dialysis and ii) with newly diagnosed ESRD from the meta-analysis on all-cause mortality and hospitalization due to influenza or pneumonia.

We evaluated the presence of residual confounding by contrasting estimates of VE measured during the influenza season to VE estimates measured during “control periods” outside the influenza season in the same studies (so-called “pseudo-effectiveness”). This approach makes the assumption that vaccination is effective against influenza-related outcomes only during the influenza season when influenza virus is circulating. Consequently, all differences in outcomes between vaccinated and non-vaccinated participants measured outside the influenza season cannot be attributed to vaccination, but must be due to other factors which differ between groups. The aim of adjustment for confounders in statistical analyses is to eliminate the influence of such factors. Residual confounding was therefore defined as present if the confounder-adjusted estimate showed a statistically significant effect of vaccination on a given outcome in the absence of virus circulation, i.e., during a control period.

Formal testing for publication bias was not performed because of the small number of identified studies. Calculations were done using STATA 12 (StataCorp, College Station, TX, USA) and RevMan 5.2 (Cochrane Collaboration). GRADE evidence profiles were created using the GRADEpro software (GRADE working group).

## Results

### Selection of studies and study characteristics

**Figure 1:**
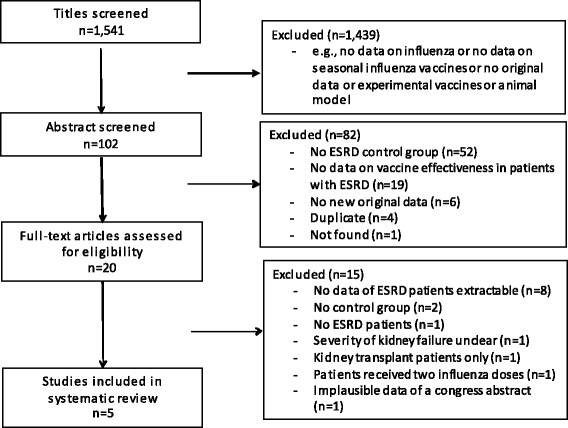
**Flow chart for the systematic literature search and study selection related to influenza vaccine efficacy/effectiveness outcomes in patients with end-stage renal disease.**

We identified, in the initial search, 1,541 records in electronic databases (Figure 1) and finally included a total of five studies in this review after applying the inclusion and exclusion criteria. Details on the excluded studies are reported in Additional file 2.

All included studies were retrospective cohort studies [23-27]. No RCTs, experimental, or quasi-experimental studies were identified. Four studies were conducted in the US and one in Taiwan; Table 1 presents the baseline characteristics of the studies. All four studies from the US identified patients with ESRD through dialysis facility networks (US Renal Data System, USRDS) during different influenza seasons [24-27]. The study from Taiwan used data from a National Health Insurance program. Three studies included patients on hemodialysis only [23,26,27], and the other two studies [24,25] comprised patients on hemodialysis and patients on peritoneal dialysis. In all studies, the main cause of ESRD was underlying diabetes mellitus. Two studies provided data on the proportion of patients with previous kidney transplantation [23,27] and one study censored patients at the time of transplantation [26].

**Table 1:** **Characteristics of included studies on influenza vaccine effectiveness in patients with end-stage renal disease**

**Author, country [Ref]**	**Study design, year**	**Age in years**	**Male patients (%)**	**Years on dialysis**	**Patients on hemodialysis (%)**	**Diagnosis of renal disease, identification of patients**	**Predominantly circulating influenza strains**	**Study size**
		**(mean ± SD or range)**		**(mean ± SD or range)**				
Bond et al., US [25]	Cohort, 2005–2006	vacc.: 60.6 ± 15.2	vacc.: 50.8	vacc.: 4.5 ± 3 .6	vacc.: 92.6	USRDS; ICD-9-CM codes	Not reported	vacc.: n = 14,226
		unvacc.: 57.9 ± 15.9	unvacc.: 52.5	unvacc.: 4.8 ± 4.1	unvacc.: 90.4			unvacc.: n = 5,994
Gilbertson et al., US [24]^1^	Cohort, 1997–1999	vacc.: 40–64: 36.3%; 65+: 53.8%	vacc.: 40.3	Proportion > 4 yrs on dialysis:	0^1^	USRDS; ICD-9-CM codes^1^	Not reported	Patients on peritoneal dialysis: n = 13,091
		unvacc.: 40–64: 64.7%; 65+: 46.2%	unvacc.: 59.7	vacc: 35.9%				
				unvacc.: 37.7%				
McGrath et al., US [26]	Cohort, 1997–1999, 2001	vacc.: 62.3–63.9^2^	vacc.: 52.2-53.0^2^	Proportion > 4 yrs on dialysis:	100	USRDS; ICD-9-CM codes	H3N2 (all seasons)	vacc.: n = 52,287–61,800^2^
		unvacc.: 60.3–61.7^2^	unvacc.: 50.4-51.6^2^	vacc.: 29.2-30.9%^2^				unvacc.: n = 55,178–64,899^2^
				unvacc.: 32.5-34.1%^2^				
Slinin et al., US [27]	Cohort, 1993–1994	all: 60.3	all: 51.1	Proportion < 5 yrs on dialysis:	100	USRDS; ICD-9-CM codes	Not reported	all: n = 10,635
				all: 75.8%				
Wang et al., Taiwan [23]	Cohort, 1998–2009	vacc.: 70.2 ± 9.96	vacc.: 50.3	Patients with “newly diagnosed” ESRD	100	Universal insurance data, National Health Insurance program	Not reported	vacc.: n = 831 unvacc.: n = 3,187
		unvacc.: 59.4 ± 14.5	unvacc.: 48.7					
**Total study population**								Total: n = 174,663^3^

ESRD, end-stage renal disease; vacc., vaccinated; unvacc., non-vaccinated.

^1^Since Gilbertson et al. and McGrath et al. used the same database, but McGrath included patients on hemodialysis only, patients on peritoneal dialysis were extracted from the study of Gilbertson et al.

^2^Range over 4 seasons.

^3^Since all US studies used the same database, overlapping of the populations cannot be ruled out.

Two US studies reported data of the same influenza season [24,26]. To avoid analyzing data from overlapping populations, we decided to analyze data from hemodialysis patients from the more recent publication [26] and those from patients on peritoneal dialysis from the older publication [24].

### Reported outcomes

Overall, nine different clinical outcomes were reported (Table 2). All-cause mortality, all-cause hospitalization, and hospitalization due to influenza or pneumonia were addressed by at least two studies, whereas the remaining outcomes were reported by one study each.

**Table 2:** **Outcomes reported in the included studies on influenza vaccine effectiveness in patients with end-stage renal disease**

**Author [Ref]**	**All-cause mortality**	**All-cause hospitalization**	**Hospitalization due to influenza or pneumonia**	**Hospitalization due to other reasons**	**Influenza-like illness**	**ICU admission**	**Estimates outside influenza season**	**Safety**
Bond et al. [25]	+	–	–	–	–	–	+	–
Gilbertson et al. [24]	+^1^	+	+	+^2^	–	–	–	–
McGrath et al. [26]	+	–	+	–	+	–	+	–
Slinin et al. [27]	–	–	+	–	–	–	–	–
Wang et al. [23]	+	+	+	+^3^	–	+	–	–

^1^Additional outcomes reported: cardiac death, death through infection.

^2^Additional outcomes reported: hospitalization due to bacteremia/viremia/septicemia; hospitalization due to respiratory infection.

^3^Additional outcomes reported: hospitalization due to bacteremia/viremia/septicemia; hospitalization due to heart disease; hospitalization due to respiratory failure.

For the assessment of risk of bias, two studies measured “pseudo-effectiveness” of influenza-related outcomes outside influenza seasons [25,26]. From the article of Bond et al. [25] we extracted off-season estimates calculated for the months June to August; from the article McGrath et al. [26] we used off-season estimates that were calculated for the pre-influenza period, when, according to the national influenza surveillance data, less than 10% of isolates were positive for influenza. In addition, one study compared vaccinated patients in vaccine-well-matched years with those in mismatched years [26]. The latter study exploits the year-to-year variation of match of the vaccine virus to the circulating wild virus strain and assumes that vaccination was effective only during seasons with a good match, whereas it had only minimal effect during mismatched seasons. In season 1997/1998, which was covered by this study, circulating A(H3N2) influenza strains did not match the vaccine strain [28,29].

### Risk of bias assessment in individual studies

Risk of bias assessment of the included studies is shown in Table 3. In four studies, risk of bias was high regarding all reported outcomes [23,24,26,27], and was mainly influenced by missing baseline data of the vaccinated vs. unvaccinated cohort [24,27] and insufficient controlling for confounders, as, for example, indicated by significant VE estimates during “control periods” or during “mismatched” seasons (see above) [26]. In one study, risk of bias was unclear [25] owing to self-reported vaccination status.

**Table 3:** **Pooled crude and adjusted odd ratios (OR) for influenza-related outcomes during influenza-season and off-season in vaccinated vs. non-vaccinated end-stage renal disease participants**

**Outcome**	**Author [Ref]**	**Crude OR (95% CI)**	**Adjusted OR (95% CI)**	**Off-season adjusted OR (95% CI)**	**Risk of bias**
**Mortality**					
**All-cause mortality**					
	Bond et al.^1^ [25]	0.79 (0.72–0.87)	0.73 (0.67–0.81)^2^	0.90 (0.77–1.10)^2^	Unclear
	Gilbertson^3^ [24]	–	0.77 (0.65–0.90)^4^		High
	McGrath [26]	0.77 (0.76–0.78)^5^	0.71 (0.70–0.72)^5^	0.45 (0.41–0.50)^6^	High
	Wang [23]	0.88 (0.73–1.07)^7^	0.49 (0.41–0.59)^7^	–	High
	**Pooled estimate**	0.77 (0.75–0.80), I^2^ = 10%	0.68 (0.61–0.76), I^2^ = 83%	–	–
**Cardiac death** ^**#**^					
	Gilbertson^3^ [24]	–	0.84 (0.71–0.98)^4^	–	High
**Infectious death** ^**§**^					
	Gilbertson^3^ [24]	–	0.83 (0.65–1.05)^4^	–	High
**Hospitalization**					
**All-cause hospitalization**					
	Gilbertson^3^ [24]	–	0.95 (0.85–1.07)^4^		High
	Wang [23]	1.11 (0.96–1.28)^7^	0.80 (0.69–0.94)^7^	–	High
	**Pooled estimate**	–	0.88 (0.74–1.04), I^2^ = 70%	–	–
**Hospitalization due to influenza or pneumonia**					
	Gilbertson^3^ [24]	–	0.90 (0.70–1.16)^4^		High
	McGrath [26]	0.90 (0.87–0.92)^5^	0.84 (0.82–0.84)^5^	0.74 (0.64–0.85)^6^	High
	Slinin [27]	–	0.93 (0.86–1.01)	–	High
	Wang [23]	1.30 (1.08–1.56)^7^	0.77 (0.64–0.93)^7^	–	High
	**Pooled estimate**	1.07 (0.75–1.53), I^2^ = 93%	0.86 (0.80–0.93), I^2^ = 58%		–
**Hospitalization due to bacteremia, viremia, or septicemia**					
	Gilbertson^3^ [24]	–	0.73 (0.32–1.68)^4^	–	High
**Hospitalization due to respiratory infection**					
	Gilbertson^3^ [24]	–	0.87 (0.69–1.09)^4^	–	High
**ICU admission**					
	Wang [23]	0.38 (0.27–0.53)^7^	0.19 (0.14–0.27)^7^	–	High
**Other outcomes**					
**Influenza-like illness**					
	McGrath [26]	0.93 (0.91–0.95)^5^	0.88 (0.86–0.90)^5^	0.77 (0.68–0.88)^6^	High

OR, odds ratio; ICU, intensive care unit.

^#^Cardiac death, defined according to cause of death reported on the ESRD death notification form (myocardial infarction, pericarditis, atherosclerotic heart disease, cardiomyopathy, cardiac arrhythmia, cardiac arrest, valvular heart disease, pulmonary edema).

^#^Infectious death, defined according to cause of death reported on the ESRD death notification form (septicemia, pulmonary infection, viral infection, tuberculosis, hepatitis B, other viral hepatitis, fungal peritonitis, other infections).

^1^OR were also reported for those who additionally received pneumococcal vaccine; however, for the purpose of this study these patients were not considered.

^2^Off-season estimates in months June–August.

^3^Only patients on peritoneal dialysis.

^4^Point estimates of two influenza seasons were pooled first.

^5^Point estimates of four seasons were pooled first.

^6^Point estimates of four pre-influenza-seasons (defined as 10% of isolates positive for influenza) were pooled first.

^7^Crude/Adjusted incidence rate ratios.

### Vaccine effectiveness and vaccine safety

**Figure 2:**
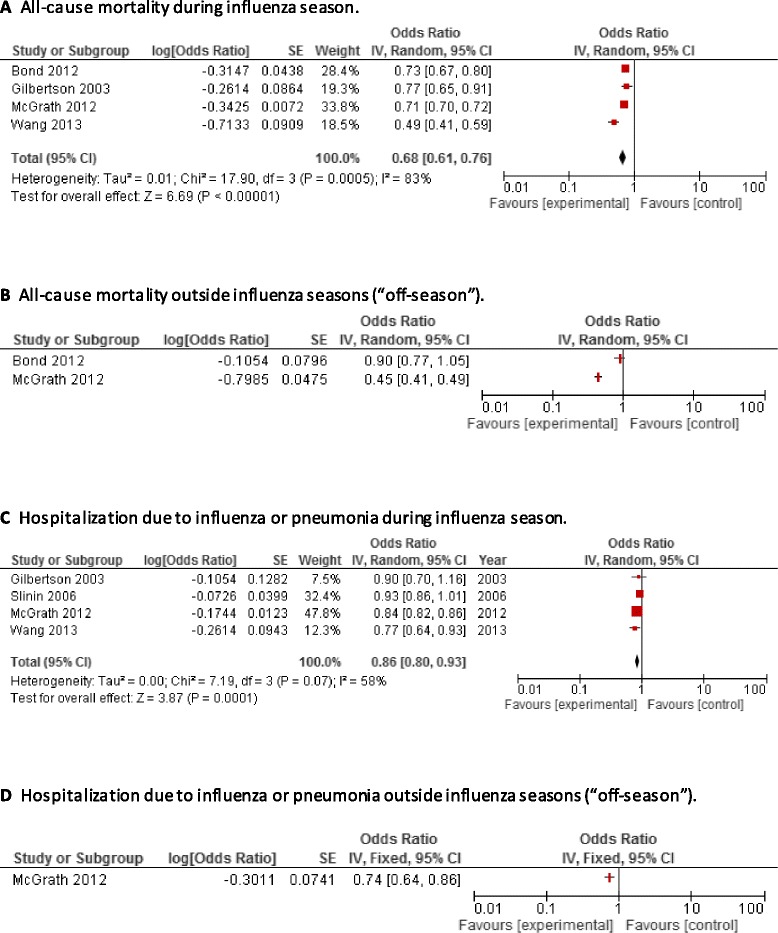
**Forest plots of observational studies presenting data on vaccine effectiveness in patients with end-stage renal disease. (A)** Adjusted effectiveness of influenza vaccination against all-cause mortality, influenza season; **(B)** Adjusted effectiveness of influenza vaccination against all-cause mortality, off-season; **(C)** Adjusted effectiveness of influenza vaccination hospitalization due to influenza or pneumonia, influenza season; **(D)** Adjusted effectiveness of influenza vaccination hospitalization due to influenza or pneumonia, off-season.

Crude and adjusted ORs for all reported outcomes are shown in Table 3. Forest plots are shown in Figure 2 and variables that were used in the final multivariate model are reported in Additional file 3. Regarding mortality, statistically significant estimates indicating a protective effect of vaccination were found for all-cause mortality (pooled confounder-adjusted VE, 32%; 95% CI, 24–39%; I^2^ = 83%; NNV: 15) and cardiac death (adjusted VE, 16%; 95% CI, 2–29%; NNV: 125), but not for death due to infection (adjusted VE, 17%; 95% CI, −5%–35%) (for definition of outcomes, see footnotes in Table 3). However, one of two studies [26] showed a significant protective effect of vaccination for all-cause mortality also in the absence of influenza virus circulation (VE, 55%; 95% CI, 50–59%), indicating residual confounding. The other study that provided a point estimate during the off-season showed no statistically significant effects [25].

Regarding hospitalization, significant protective effects of influenza vaccination were observed for the outcomes hospitalization due to influenza or pneumonia (pooled adjusted VE, 14%; 95% CI, 7–20%; I^2^ = 58%; NNV: 42) and ICU admission (adjusted VE, 81%; 95% CI, 63–86%; NNV: 18). No significant effects were found for all-cause hospitalization (pooled adjusted VE, 12%; 95% CI, −6–26%; I^2^ = 70%), hospitalization due to bacteremia, viremia, or septicemia (adjusted VE, 27%; 95% CI, −32–68%) and hospitalization due to respiratory infection (adjusted VE, 13%; 95% CI, −9–31%). A significant off-season estimate indicated the presence of residual confounding for the effect on hospitalization due to influenza or pneumonia (adjusted off-season VE, 26%; 95% CI, 15–36%). Likewise, effect on influenza-like illness was likely to be prone to residual confounding, indicated by a significant off-season VE which was even higher than the in-season VE (Table 3).

The sensitivity analysis revealed that the removal of data of patients on peritoneal dialysis [24] or of those with newly diagnosed ESRD [23] did not affect point estimates significantly (Additional file 4).

Laboratory-confirmed influenza infections were not reported. None of the studies provided data on vaccine safety, neither on local nor on systemic adverse events.

### Quality of evidence

The body of evidence on influenza VE regarding all reported outcomes was rated as being of very low quality due to serious risk of bias (see Table 4 for GRADE evidence profile). Since data on vaccine safety could not be extracted, no rating of evidence quality could be performed.

**Table 4:** **GRADE evidence profile for effectiveness of influenza vaccination in patients with end-stage renal disease**

**Quality assessment**							**No of patients**		**Effect**		**Quality**	**Importance**
**No of studies**	**Design**	**Risk of bias**	**Inconsistency**	**Indirectness**	**Imprecision**	**Other considerations**	**Vaccination against influenza**	**Control**	**Relative (95% CI)**	**Absolute**		
**All-cause mortality**												
4	Observational studies	Serious^1^	No serious inconsistency	No serious indirectness	No serious imprecision	None	–	1,798/8,759 (20.5%)^2^	RR 0.68 (0.61–0.76)^3^	66 fewer per 1,000 (from 49 fewer to 80 fewer)	⊕ΟΟΟ VERY LOW	CRITICAL
								10%		32 fewer per 1,000 (from 24 fewer to 39 fewer)		
								40%		128 fewer per 1,000 (from 96 fewer to 156 fewer)		
**Cardiac death**												
1	Observational studies	Serious^4^	No serious inconsistency	No serious indirectness	No serious imprecision	None	–	5%	RR 0.84 (0.71–0.98)^5^	8 fewer per 1,000 (from 1 fewer to 15 fewer)	⊕ΟΟΟ VERY LOW	CRITICAL
								10%		16 fewer per 1,000 (from 2 fewer to 29 fewer)		
								20%		32 fewer per 1,000 (from 4 fewer to 58 fewer)		
**Infectious death**												
1	Observational studies	Serious^4^	No serious inconsistency	No serious indirectness	No serious imprecision	None	–	5%	RR 0.83 (0.65–1.05)^5^	9 fewer per 1,000 (from 18 fewer to 2 more)	⊕ΟΟΟ VERY LOW	CRITICAL
								10%		17 fewer per 1,000 (from 35 fewer to 5 more)		
								20%		34 fewer per 1,000 (from 70 fewer to 10 more)		
**All-cause hospitalization**												
2	Observational studies	Serious^1^	No serious inconsistency	No serious indirectness	No serious imprecision	None	–	1,688/1,888 (89.4%)^6^	RR 0.88 (0.74–1.04)^7^	107 fewer per 1,000 (from 232 fewer to 36 more)	⊕ΟΟΟ VERY LOW	CRITICAL
								20%		24 fewer per 1,000 (from 52 fewer to 8 more)		
								40%		48 fewer per 1,000 (from 104 fewer to 16 more)		
**Influenza/pneumonia hospitalization**												
4	Observational studies	Serious^8^	No serious inconsistency	No serious indirectness	No serious imprecision	None	–	445/2,584 (17.2%)	RR 0.86 (0.8–0.93)^3^	24 fewer per 1,000 (from 12 fewer to 34 fewer)	⊕ΟΟΟ VERY LOW	CRITICAL
								5%		7 fewer per 1,000 (from 3 fewer to 10 fewer)		
								30%		42 fewer per 1,000 (from 21 fewer to 60 fewer)		
**Hospitalization due to bacteremia, viremia or septicemia**												
1	Observational studies	Serious^4^	No serious inconsistency	No serious indirectness	No serious imprecision	None	–	5%	RR 0.73 (0.32–1.68)^5^	13 fewer per 1,000 (from 34 fewer to 34 more)	⊕ΟΟΟ VERY LOW	CRITICAL
								10%		27 fewer per 1,000 (from 68 fewer to 68 more)		
								20%		54 fewer per 1,000 (from 136 fewer to 136 more)		
**Hospitalization due to respiratory infection**												
1	Observational studies	Serious^4^	No serious inconsistency	No serious indirectness	No serious imprecision	None	–	5%	RR 0.87 (0.69–1.09)^5^	6 fewer per 1,000 (from 16 fewer to 5 more)	⊕ΟΟΟ VERY LOW	CRITICAL
								10%		13 fewer per 1,000 (from 31 fewer to 9 more)		
								20%		26 fewer per 1,000 (from 62 fewer to 18 more)		
**ICU admission**												
1	Observational studies	Serious^9^	No serious inconsistency	No serious indirectness	No serious imprecision	None	–	184/2,696 (6.8%)	RR 0.19 (0.14–0.27)	55 fewer per 1,000 (from 50 fewer to 59 fewer)	⊕ΟΟΟ VERY LOW	CRITICAL
								12%		97 fewer per 1,000 (from 88 fewer to 103 fewer)		
								25%		203 fewer per 1,000 (from 183 fewer to 215 fewer)		
**Influenza-like illness**												
1	Observational studies	Serious^10^	No serious inconsistency	No serious indirectness	No serious imprecision	None	–	5%	RR 0.88 (0.86–0.9)^11^	6 fewer per 1,000 (from 5 fewer to 7 fewer)	⊕ΟΟΟ VERY LOW	CRITICAL
								10%		12 fewer per 1,000 (from 10 fewer to 14 fewer)		
								20%		24 fewer per 1,000 (from 20 fewer to 28 fewer)		

^1^High risk of bias in two of four studies due to inappropriate adjustment for confounders and unclear baseline imbalance.

^2^Control group rates available in only two of four studies.

^3^RR adjusted in all four studies at least for age, sex, and comorbidities.

^4^High risk of bias due to missing information on comorbidities in vaccinated vs. non-vaccinated participants.

^5^Adjusted for age, sex, ethnicity, network, length of time with ESRD, cause of renal failure, comorbidity index, and hospital days.

^6^Control group rate available only for one of two studies.

^7^RR adjusted in both studies at least for age, sex, and comorbidities.

^8^High risk of bias in three of four studies due to inappropriate adjustment for confounders, unclear baseline imbalance, and inappropriate follow-up time.

^9^High risk of bias due to inappropriate adjustment for confounders.

^10^Significant estimate of effectiveness outside influenza season indicates residual confounding.

^11^Adjusted for sex, age, cause of ESRD, vintage, adherence, hospital days, mobility aids, network, comorbidities, and oxygen use.

## Discussion

Our results indicate that there is only very low quality evidence that influenza vaccination of patients with ESRD can prevent mortality, hospitalization, or other clinical outcomes. Although pooled estimates showed small to moderate protective effects against all-cause mortality and hospitalization due to influenza or pneumonia in this patient sub-group, VE that was measured outside influenza seasons showed even greater protective effects, thereby strongly indicating residual confounding. Protective effects against other clinical outcomes were either not statistically significant or only reported by single studies with a high risk of bias.

There are a few immunogenicity studies published that suggested that ESRD patients might have an impaired immune-response to inactivated influenza vaccines [14]. However, other studies showed contradicting results and it remains unclear how well seroprotection rates translate into protection against clinical outcomes in general and how well humoral response is sufficient for protection in this patient sub-group in particular [15,16]. Recently, the European Medicines Agency has changed its policy in the approval of seasonal influenza vaccines and has withdrawn the “*Note for Guidance on Harmonisation of Requirements for Influenza Vaccines (CPMP/BWP/214/96)*” [30]. According to the European Medicines Agency, post-marketing studies monitoring the clinical benefit and risk profile of seasonal influenza vaccines should be strengthened, whereas providing immunogenicity data from small clinical trials should no longer be conducted, since these data might not correlate to the expected efficacy and safety of the vaccine [31]. These arguments highlight the need to critically evaluate and summarize the available evidence by focusing on clinical outcomes rather than using surrogate markers of vaccine effectiveness. However, our review also shows the challenges that are related with the conduct of observational studies on influenza VE and when making decisions on regulatory aspects or vaccine recommendations based on only low or very low quality of evidence.

In our study, pooled VE estimates against all-cause mortality and hospitalization due to pneumonia and influenza were derived from four studies. If they were free of bias and confounding, they would indicate protective, albeit small to moderate effects in patients with ESRD. Given the high rates of respective events in this at-risk group, even a low effectiveness of 32% and 14% against all-cause mortality and hospitalization due to influenza or pneumonia, respectively, would correspond to a NNV of 15 and 42, respectively. Although differences between ESRD subpopulations are likely as, for example, shown in one study [24] by statistical significant differences in baseline characteristics between patients on hemodialysis and those on peritoneal dialysis, removal of patients on peritoneal dialysis or with newly diagnosed ESRD from the meta-analysis did not affect point estimates significantly. This was due to the large power of the study by McGrath et al. [26], which mainly influenced the results. Therefore, further conclusions from this sensitivity analysis have to be taken with caution.

When interpreting the VE results on clinical outcomes reported here, several issues have to be taken into account. First, risk of bias was high in four studies and unclear in the remaining one. This was due to inappropriate adjustment for confounders, unclear baseline imbalances of vaccinated and unvaccinated subpopulations, or strong indicators of residual confounding such as significant or even stronger protective vaccine effects outside influenza seasons. Second, since unspecific outcomes tend to ‘dilute’ the effectiveness of (influenza) vaccines [32], it remains unclear why VE was found to be higher for all-cause mortality than for the more specific outcome influenza/pneumonia hospitalization.

Interestingly, from a methodological perspective, was the approach used by McGrath et al. [26] when comparing VE in a year when the vaccine strains did not match with the circulating strain (unmatched season) with VE during a well-matched year. This approach can be used as an additional or alternative strategy to the “pseudo-effectiveness approach outside seasons” to assess the risk of bias in observational studies on influenza VE. Using the unmatched season as ‘working placebo’ did not reveal a protective effect of influenza vaccination against any clinical outcome. The authors concluded that the potential benefit of the influenza vaccine in patients with ESRD is small to negligible and protective effects measured using the conventional approach are likely to be biased. This issue might be driven by the ‘healthy vaccine effect’ and has been discussed previously for influenza vaccines [33]. It has been suggested that estimating off-season estimates in observational studies could be helpful to assess the extent of unmeasured confounding [34]. However, a recently conducted study found that even adjusting for more than 100 variables did not eliminate unmeasured confounding and that, instead, using the instrumental variable analysis method is effective in producing less-biased estimates [35]. Furthermore, outcome misclassification could be prevented by using laboratory-confirmed influenza as an outcome, rather than unspecific indicators of mortality and morbidity.

Remarkably, we did not identify studies that compared safety outcomes between vaccinated and unvaccinated patients with ESRD. In healthy adults, vaccination against seasonal influenza is not associated with an increased risk of serious adverse events [36,37]. However, in patients with ESRD, studies comparing reactogenicity of two different influenza vaccines [38] (subunit vs. virosomal) or assessing the immunogenicity among vaccinated participants [39,40] did not show any serious adverse events. In addition, two studies did not find differences in adverse event rates after influenza vaccination between patients with ESRD compared with healthy adults [41,42]. Although limited by the lack of control groups and sample size, there is no clear evidence of an increased risk of severe adverse events following influenza vaccination in patients with ESRD.

Our study has several strengths. It is the first systematic review on this topic, covering all data published so far. In addition, by searching the largest respective data base (ClinicalTrials.gov) we also aimed at identifying unpublished studies. We performed an outcome-specific quality assessment of individual studies and considered the quality of the body of evidence for each outcome by using GRADE. The limitations of our systematic review were mainly due to the limitations of the included studies. Surprisingly, we identified studies from two countries only, although the number of patients with ESRD requiring dialysis is increasing worldwide [1-3]. Four of five included studies were conducted in the US, using the same data source. The USRDS database comprises the largest population of dialysis patients worldwide; however, the composition of this population might differ substantially from ESRD patients from other countries. Moreover, the USRDS is based on administrative claims data and some important variables, including the vaccination exposure might have not been adequately captured. For example, three studies [24-26] assessed the potential of exposure misclassification: Bond et al. [25] reported that, in a subsample, vaccination was reported by patients themselves and was not validated through clinical records. However, the authors argued that the mortality rate was higher among patients with self-reported (compared to database-documented) vaccination status and that therefore incorrect self-reports of having received vaccination would have biased the results towards the null. Gilbertson et al. [24] concluded that the low vaccination rate observed in their study might reflect a low sensitivity of the billing data that was used to determine vaccination status but that interpretation here is difficult. McGrath et al. [26] assumed that the number of vaccinations missed (e.g., if patients received a vaccine that was paid out of pocket) would be low given the fact that the vaccine is paid by the health maintenance organization covering the study population. In addition, at least partial overlap of the study population of the four different US studies cannot be ruled out, although the studies analyzed different seasons and the outcomes should be mutually exclusive for each season. However, multiple inclusions of the same patients could have artificially equalized point estimates from different studies. Finally, since our study sample was too small to formally test for publication bias, this form of bias cannot be ruled out.

## Conclusions

In conclusion, our systematic review indicates that evidence on the protective effects of influenza vaccination in patients with ESRD is limited and of very low quality. Evidence on vaccine safety is absent. Therefore, studies using randomization or quasi-experimental designs are needed to determine the extent by which vaccination prevents influenza and related clinical outcomes in this important risk group. In addition, whether other vaccine types, such as adjuvant vaccines or high-dose vaccines, could have larger effects in this population compared to trivalent inactivated vaccines should also be assessed. Given the high rates of health-endangering events in these patients, even a low VE can be considered as sufficient to recommend annual influenza vaccination. However, physicians should consider influenza in their differential diagnosis if ESRD patients present with influenza-like illness symptoms regardless of whether they are vaccinated or not.

## Additional files

Additional file 1:
**Search strategy details.**
Additional file 2:
**List of excluded studies.**
Additional file 3:
**Variables considered in the final adjusted analysis.**
Additional file 4:
**Sensitivity analysis on adjusted effectiveness of influenza vaccination.**

## Abbreviations

CI: Confidence interval
ESRD: End-stage renal disease
GRADE: Grading of Recommendations Assessment Development and Evaluation
HR: Hazard ratio
NNV: Number needed to vaccinate
OR: Odds ratio
RCT: Randomized controlled trial
RD: Risk difference
RR: Relative risk
USRDS: US Renal Data System
VE: Vaccine effectiveness
