# Supplementary material for: Influenza vaccination in patients with end-stage renal disease: systematic review and assessment of quality of evidence related to vaccine efficacy, effectiveness, and safety
Source: BMC Med. 2014 Dec 19;12:244. doi: 10.1186/s12916-014-0244-9 (PMC4298993; doi:10.1186/s12916-014-0244-9)
Supplement: Additional file 1: — Search strategy details. [file 12916_2014_244_MOESM1_ESM.docx]

**Additional file 1**

Search strategy for the systematic review on influenza vaccine efficacy, effectiveness and safety in patients with end-stage renal disease.

#1 “kidney”

#2 “dialysis”

#3 “renal”

#4 “nephr*”

#5 “hemodialysis”

#6 “haemodialysis”

#7 “uremia”

#8 #1 OR #2 OR #3 OR #4 OR #5 OR #6 OR #7

#9 “vacci*”

#10 “immuniz*”

#11 #9 OR #10

#12 “influenza”

#13 #8 AND #11 and #12

(restrictions: species: human)
