# Supplementary material for: Influenza vaccination in patients with end-stage renal disease: systematic review and assessment of quality of evidence related to vaccine efficacy, effectiveness, and safety
Source: BMC Med. 2014 Dec 19;12:244. doi: 10.1186/s12916-014-0244-9 (PMC4298993; doi:10.1186/s12916-014-0244-9)
Supplement: Additional file 2: — List of excluded studies. [file 12916_2014_244_MOESM2_ESM.docx]

**Additional file 2**

List of excluded studies on influenza vaccine effectiveness and safety in patients with end-stage renal disease (n=15):

No data of ESRD patients extractable (n=7)[[1-8](#_ENREF_1)]

No control group (n=2)[[9](#_ENREF_9), [10](#_ENREF_10)]

No ESRD patients (n=1)[[11](#_ENREF_11)]

Severity of kidney failure unclear (n=1)[[12](#_ENREF_12)]

Kidney transplant patients only (n=1)[[13](#_ENREF_13)]

Patients received two influenza doses (n=1)[[14](#_ENREF_14)]

Implausible data of a congress abstract (n=1) [[15](#_ENREF_15)]

**Excluded References**

1. Skull SA, Andrews RM, Byrnes GB, Campbell DA, Kelly HA, Brown GV, Nolan TM: **Hospitalized community-acquired pneumonia in the elderly: an Australian case-cohort study.** *Epidemiol Infect* 2009, **137:**194-202.

2. Wang CS, Wang ST, Lai CT, Lin LJ, Chou P: **Impact of influenza vaccination on major cause-specific mortality.** *Vaccine* 2007, **25:**1196-1203.

3. Voordouw AC, Sturkenboom MC, Dieleman JP, Stijnen T, Smith DJ, van der Lei J, Stricker BH: **Annual revaccination against influenza and mortality risk in community-dwelling elderly persons.** *JAMA* 2004, **292:**2089-2095.

4. Sanchez Munoz-Torrero JF, Saponi Cortes JM, Ortiz Descante C, Ojeda Garcia Escribano I, Sanchez Sanchez T, Perez Reyes F, Martin Ruiz C, Costo Campoamor A: **[Utilization and effectiveness of the flu vaccination in the prevention of the hospitalization induced by cardiorespiratory decompensation in high-risk patients in Caceres].** *Rev Clin Esp* 2003, **203:**363-367.

5. Nichol KL, Wuorenma J, von Sternberg T: **Benefits of influenza vaccination for low-, intermediate-, and high-risk senior citizens.** *Arch Intern Med* 1998, **158:**1769-1776.

6. Castilla J, Moran J, Martinez-Artola V, Reina G, Martinez-Baz I, Garcia Cenoz M, Alvarez N, Irisarri F, Arriazu M, Elia F, Salcedo E: **Effectiveness of trivalent seasonal and monovalent influenza A(H1N1)2009 vaccines in population with major chronic conditions of Navarre, Spain: 2010/11 mid-season analysis.** *Euro Surveill* 2011, **16**.

7. Ritzwoller DP, Bridges CB, Shetterly S, Yamasaki K, Kolczak M, France EK: **Effectiveness of the 2003-2004 influenza vaccine among children 6 months to 8 years of age, with 1 vs 2 doses.** *Pediatrics* 2005, **116:**153-159.

8. Yoshimoto T: **[Studies on the prophylactic effect of influenza vaccine. Antibody response and side reactions in patients under treatment by hemodialysis].** *Nihon Ika Daigaku Zasshi* 1985, **52:**178-185.

9. Krairittichai U, Chittaganpitch M: **Efficacy of the trivalent influenza vaccination in Thai patients with hemodialysis or kidney transplant compared with healthy volunteers.** *J Med Assoc Thai* 2013, **96 Suppl 3:**S1-7.

10. Wyzgal J, Brydak LB, Zygier D, Paczek L, Rowinski W, Grochowiecki T: **Study on efficacy of influenza vaccination in renal allograft recipients.** *Transplant Proc* 2002, **34:**572-575.

11. Chan TC, Yap YH, Hung FN, Shea YF, Chu LW, Luk KH, Woo CY, Chan HW: **The efficacy of influenza vaccination is reduced in nursing home older adults with moderate to severe renal impairment.** *J Am Med Dir Assoc* 2013, **14:**133-136.

12. Gasparini R, Amicizia D, Lai PL, Rossi S, Panatto D: **Effectiveness of adjuvanted seasonal influenza vaccines (Inflexal V (R) and Fluad (R) ) in preventing hospitalization for influenza and pneumonia in the elderly: a matched case-control study.** *Hum Vaccin Immunother* 2013, **9:**144-152.

13. Hurst FP, Lee JJ, Jindal RM, Agodoa LY, Abbott KC: **Outcomes associated with influenza vaccination in the first year after kidney transplantation.** *Clin J Am Soc Nephrol* 2011, **6:**1192-1197.

14. Dogliani M, Fidelio T, Scalzo B, Iacono G, Deabate MC, Bagatella M, Saracco B: **[Effectiveness of influenza vaccination in patients undergoing regular dialysis treatments].** *Minerva Urol Nefrol* 1997, **49:**121-124.

15. Trajceska L, Severova G, Dzekova P, Amitov D, Mladenovska D, Gelev S, Selim G, Sikole A: **Clinical effectiveness of vaccination against influenza in dialysis patients. Poster No 77. ESAO (European Society for Artificial Organs) congress. Rostock, Germany, September 26th – 29th, 2012. Available at:** [**http://www.esao2012.org/index.php/programme/programm-overview/friday-0928/poster-session-2-p65-p83**](http://www.esao2012.org/index.php/programme/programm-overview/friday-0928/poster-session-2-p65-p83)**. Accessed on 1 June 2014.** 2012.
