# Supplementary material for: Influenza vaccination in patients with end-stage renal disease: systematic review and assessment of quality of evidence related to vaccine efficacy, effectiveness, and safety
Source: BMC Med. 2014 Dec 19;12:244. doi: 10.1186/s12916-014-0244-9 (PMC4298993; doi:10.1186/s12916-014-0244-9)
Supplement: Additional file 3: — Variables considered in the final adjusted analysis. [file 12916_2014_244_MOESM3_ESM.docx]

**Additional file 3**

Variables considered in the final adjusted analysis in the included studies reporting on influenza vaccine effectiveness and safety in patients with end-stage renal disease (ESRD).

| **Study** | **Variables considered in the final adjusted analysis** |
| --- | --- |
| Bond et al. | Age, race, sex, time on dialysis, modality (hemodialysis, continuous cyclic or ambulatory peritoneal dialysis), diabetes as primary cause of ESRD, comorbidities at dialysis initiation, and laboratory parameters. |
| Gilbertson et al. | Age, sex, race, ethnicity, ESRD network, length on time with ESRD, cause of renal failure, Charlson comorbidity index, and severity of disease measure of hospital days during the eight-month entry period. |
| McGrath et al. | Age, race, sex, cause of ESRD, vintage, adherence, hospital days, mobility aids, ESRD network, comorbidities, and oxygen use. |
| Slinin et al. | Age, race, sex, smoking status, duration of dialysis, cause of ESRD, previous transplantation, body mass index, laboratory parameters, and comorbidities. |
| Wang et al. | Age, sex, comorbidities, and calendar years. |
