# Supplementary material for: Influenza vaccination in patients with end-stage renal disease: systematic review and assessment of quality of evidence related to vaccine efficacy, effectiveness, and safety
Source: BMC Med. 2014 Dec 19;12:244. doi: 10.1186/s12916-014-0244-9 (PMC4298993; doi:10.1186/s12916-014-0244-9)

**Additional file 4**

Sensitivity analysis on adjusted effectiveness of influenza vaccination against (1) all-cause mortality and (2) hospitalization due to influenza or pneumonia during influenza season in patients with end-stage renal disease (ESRD).

**(1)** All-cause mortality: **1A**. all studies; **1B**. without patients on peritoneal dialysis (Gilbertson et al.); **1C**. without patients with newly diagnosed ESRD (Wang et al.); **1D**. without patients on peritoneal dialysis or with newly diagnosed ESRD.

**1A**. All-cause mortality during influenza season, all studies.


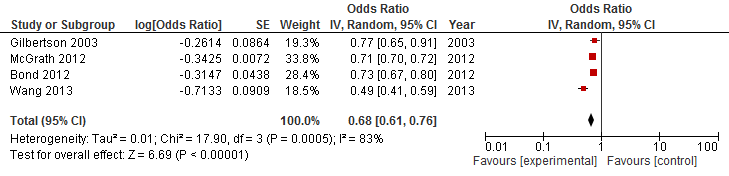


**1B**. All-cause mortality during influenza season, without patients on peritoneal dialysis (Gilbertson et al.).
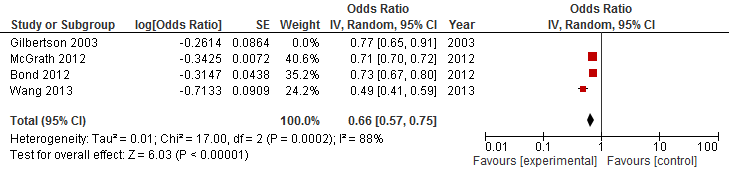


**1C**. All-cause mortality during influenza season, without patients with newly diagnosed ESRD (Wang et al.).
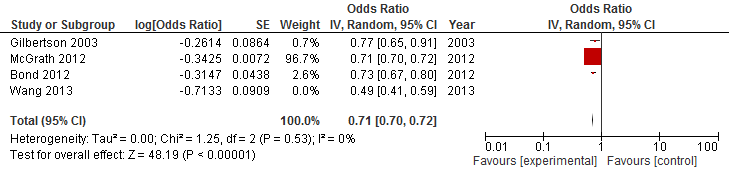


**1D**. All-cause mortality during influenza season, without patients on peritoneal dialysis or with newly diagnosed ESRD.


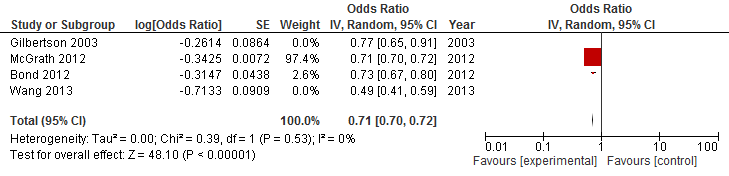


**(2)** Hospitalization due to influenza or pneumonia: **2A**. all studies; **2B**. without patients on peritoneal dialysis (Gilbertson et al.); **2C**. without patients with newly diagnosed ESRD (Wang et al.); **2D**. without patients on peritoneal dialysis or with newly diagnosed ESRD.

**2A**. Hospitalization due to influenza or pneumonia during influenza season, all studies.


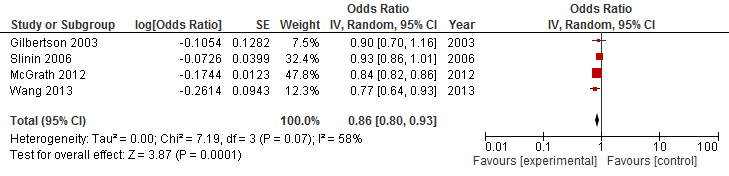


**2B**. Hospitalization due to influenza or pneumonia during influenza season, without patients on peritoneal dialysis (Gilbertson et al.).


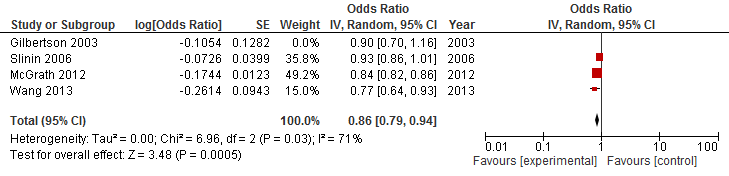


**2C**. Hospitalization due to influenza or pneumonia during influenza season, without patients with newly diagnosed ESRD (Wang et al.).


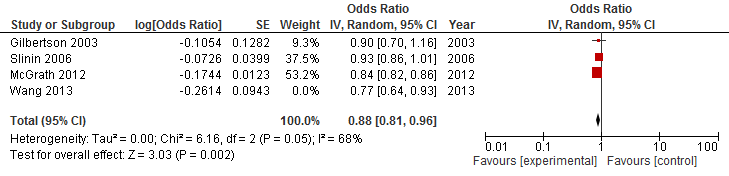


**2D**. Hospitalization due to influenza or pneumonia during influenza season, without patients on peritoneal dialysis or with newly diagnosed ESRD.


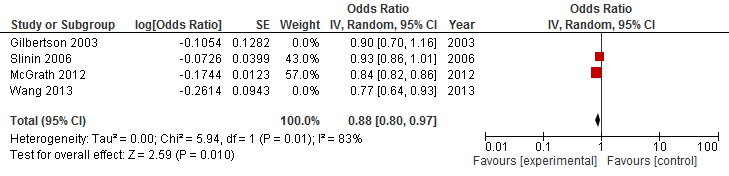

Supplement: Additional file 4: — Sensitivity analysis on adjusted effectiveness of influenza vaccination. [file 12916_2014_244_MOESM4_ESM.docx]
